# Foveal avascular zone segmentation in optical coherence tomography angiography images using a deep learning approach

**DOI:** 10.1038/s41598-020-80058-x

**Published:** 2021-01-13

**Authors:** Reza Mirshahi, Pasha Anvari, Hamid Riazi-Esfahani, Mahsa Sardarinia, Masood Naseripour, Khalil Ghasemi Falavarjani

**Affiliations:** 1 Eye Research Center, The Five Senses Institute, Rassoul Akram Hospital, Iran University of Medical Sciences, Tehran, Iran; 2 Eye Research Center, Farabi Eye Hospital, Tehran University of Medical Sciences, Tehran, Iran; 3 Stem Cell and Regenerative Medicine Research Center, Iran University of Medical Sciences, Tehran, Iran

**Keywords:** Retinal diseases, Diabetes complications, Machine learning, Image processing

## Abstract

The purpose of this study was to introduce a new deep learning (DL) model for segmentation of the fovea avascular zone (FAZ) in en face optical coherence tomography angiography (OCTA) and compare the results with those of the device’s built-in software and manual measurements in healthy subjects and diabetic patients. In this retrospective study, FAZ borders were delineated in the inner retinal slab of 3 × 3 enface OCTA images of 131 eyes of 88 diabetic patients and 32 eyes of 18 healthy subjects. To train a deep convolutional neural network (CNN) model, 126 enface OCTA images (104 eyes with diabetic retinopathy and 22 normal eyes) were used as training/validation dataset. Then, the accuracy of the model was evaluated using a dataset consisting of OCTA images of 10 normal eyes and 27 eyes with diabetic retinopathy. The CNN model was based on Detectron2, an open-source modular object detection library. In addition, automated FAZ measurements were conducted using the device’s built-in commercial software, and manual FAZ delineation was performed using ImageJ software. Bland–Altman analysis was used to show 95% limit of agreement (95% LoA) between different methods. The mean dice similarity coefficient of the DL model was 0.94 ± 0.04 in the testing dataset. There was excellent agreement between automated, DL model and manual measurements of FAZ in healthy subjects (95% LoA of − 0.005 to 0.026 mm^2^ between automated and manual measurement and 0.000 to 0.009 mm^2^ between DL and manual FAZ area). In diabetic eyes, the agreement between DL and manual measurements was excellent (95% LoA of − 0.063 to 0.095), however, there was a poor agreement between the automated and manual method (95% LoA of − 0.186 to 0.331). The presence of diabetic macular edema and intraretinal cysts at the fovea were associated with erroneous FAZ measurements by the device’s built-in software. In conclusion, the DL model showed an excellent accuracy in detection of FAZ border in enfaces OCTA images of both diabetic patients and healthy subjects. The DL and manual measurements outperformed the automated measurements of the built-in software.

## Introduction

Optical coherence tomography angiography (OCTA) is a novel noninvasive technique for depth-resolved visualization of retinal vasculature. Since the advent of OCTA, our knowledge regarding retinal microvasculature has expanded exponentially and OCTA has proved to be useful in many ischemic and non-ischemic retinal disorders including diabetic retinopathy (DR), retinal vein occlusion (RVO), and age-related macular degeneration^1^.

Several studies have reported different metrics in OCTA images for the assessment of pathologic changes. Foveal avascular zone (FAZ) area is one of the most reported OCTA metrics. Numerous studies have evaluated the changes in the FAZ area in various retinal diseases compared to healthy subjects. It has been shown that the FAZ is remodeled and enlarged in retinal vascular disorders (e.g. diabetic retinopathy) and a negative correlation exists between the FAZ area and visual acuity^2^.

Considering the importance of accurate FAZ measurements in the interpretation of the experimental and clinical studies, reliable methods should be implemented for this purpose. Currently, manual measurement and automated delineation of FAZ area by the OCTA device are the most used methods for FAZ area quantification in the literature. Reproducibility and reliability of the automated FAZ measurements using the device software in comparison to manual measurements have been previously reported in normal subjects^3–6^. However, automated FAZ measurements are less reliable in diabetic eyes and manual correction of the measurements may be required^7^.

Machine learning techniques have been used for automated diagnosis and detection of different aspects of human diseases^8^. In ophthalmology, it has been used successfully in retinal and glaucomatous disease. Deep learning has been developed as a leading machine learning tool in computer vision science and evolved to have a significant impact in the field of ophthalmic imaging. Deep learning techniques and in particular, convolutional neural networks, have rapidly gained popularity for the analysis of the retinal images.

This study aims to report the reliability of a new deep learning-based approach for the measurement of FAZ area in OCTA images and to compare the results with those of the manual FAZ segmentation and automated FAZ measurements in both healthy subjects and patients with diabetic retinopathy.

## Methods

In this retrospective comparative study, 104 eyes of 69 diabetic patients with different stages of DR and 12 eyes from 12 healthy subjects were selected for the training/validation database. Thirty-seven eyes (10 eyes from 6 normal subjects and 27 eyes from 19 diabetic patients) were used for the final evaluation of the trained system. The study was approved by the Iran University of Medical Sciences Ethics Committee (IR.IUMS.REC.1398.078) and adherents to the tenets of the declaration of Helsinki. Informed consent was obtained from all participants.

All OCTA images were obtained using RTVue XR 100 Avanti instrument (Version 2017.1.0.151, Optovue, Inc., Fremont, CA, USA). The images of patients with significant media opacity, refractive error beyond ± 3 spherical equivalent, and image quality lower than 5 were excluded from the study. The inner retinal slab, from the internal limiting membrane (ILM) to an offset of 9 µm below the outer plexiform layer (OPL), was automatically segmented in en face 3 × 3 mm OCTA images. The ILM and OPL segmentations were manually corrected if needed as described elsewhere^9,10^.

Automated FAZ measurements were performed using AngioVue, the device’s built-in commercial software. AngioVue software measures the FAZ area automatically using the “Measure: FAZ” tool. Upon detection of the FAZ area by the software, a yellow overlay delineating the border is added to the enface image of the inner retinal slab. In addition to FAZ area, signal strength of the images, presence of cystic changes in foveal center, and presence of diabetic macular edema (thickness greater than 320 µm) were recorded.

The raw OCTA images were then exported and transferred to ImageJ software (http://imagej.nih.gov/ij/; provided in the public domain by the National Institutes of Health, Bethesda, MD, USA) for manual measurements. All manual measurements were conducted by a skilled grader (RM) and rechecked by another independent grader (PA). In case of any dispute, a senior grader (KGF) corrected the outline of the FAZ area. All manual measurements were performed before running the deep learning method.

### Model training

A total of 126 enface OCTA images (104 with diabetic retinopathy and 22 healthy subjects) were used as the training dataset. The ground truth pixel labeling was based on manual segmentation of the FAZ which divided the pixels into the FAZ and non-FAZ labels.

**Figure 1:**
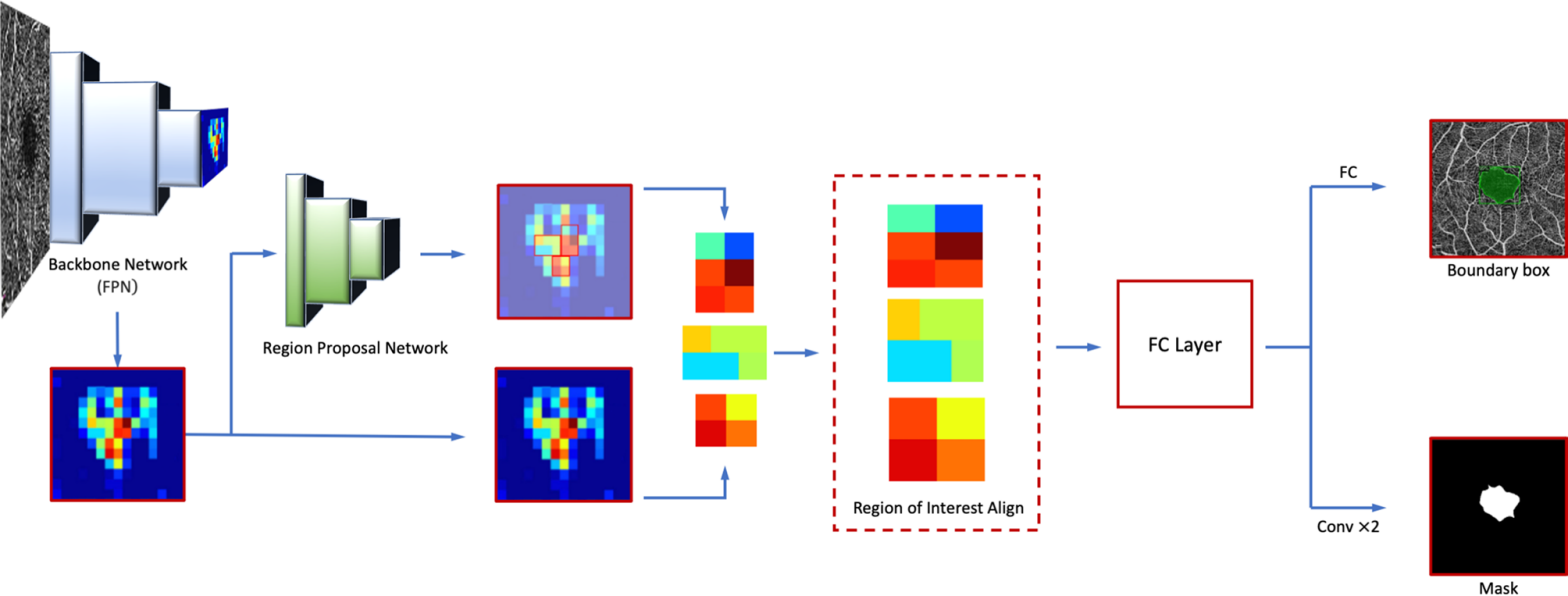
Simplified flowchart of the deep learning model simulating the steps for a single image.

Detectron2, an open-source modular object detection library developed by the Facebook AI Research (FAIR) team^11^ was used for deep learning-based image segmentation. Detectron2 is a software system that implements state-of-the-art object detection algorithms with three distinct blocks that performs semantic and instance segmentation. The first block is based on the Feature Pyramid Network (FPN)^12^ implemented in a ResNet-50 network^13^. The FPN network extracts features at predefined spatial resolutions used to construct a feature pyramid, parallel to selected feature maps in forward layers of related convolutional neural network (CNN) but containing rich semantics in all layers. In the following block, a Cascade/Mask R-CNN on top of FPN is used for segmentation. The proposed regions of interest undergo an operation called Region of Interest Align (RoIAlign) before applying Mask R-CNN to each pyramid level separately. In the final block, a lightweight dense prediction branch is used on top of the same FPN features to merge different layers into a pixel-wise output. A simplified flowchart of the model is illustrated in Fig. 1.

The pre-trained CNN on the COCO dataset^14^ was implemented using Python (version 3.6) on a cloud computing service (Google Colab). Data augmentation strategies including random flip were used for the compensation of the relatively small sample size and avoidance of model overfitting.

### Model metrics

Thirty-seven enface OCTA images of 10 normal eyes and 27 eyes with diabetic retinopathy were used as the testing dataset for validation of the model. The measured FAZ area of every subject in training and testing dataset was recorded for each individual.

For evaluation of the accuracy of instance segmentation, the predicted FAZ masks of training and test datasets were exported from the trained model. Afterwards, the Dice similarity coefficient (DSC) was calculated based on the following formula^15^:

$${\text{DSC}} = \frac{{2\left( {A \cap B} \right)}}{A + B}$$

where A is the predicted mask and B is the ground truth mask based on manual segmentation. The DSC was evaluated for each pair of images (prediction and ground truth) separately and the mean IoU across the training and testing dataset was calculated. This index is the most popular metric for showing measurement similarities in image segmentation and provides an excellent estimation of overlapping pixels between the two images.

### Statistical analysis

All statistical analyses were conducted using SPSS (IBM Corp., Armonk, NY, USA) version 22.0 and Excel 2013 (Microsoft, Redmond, WA, USA). Bland–Altman plots with 95% limit of agreement (95% LoA) was used to illustrate the agreement between the measurements. Graphpad prism version 8.0 was used for plotting Bland–Altman graphs. In addition, the correlation coefficient was calculated for evaluation of consistency in FAZ measurements. To address the inter-eye correlation for the enrolled bilateral cases, the generalized estimating equation (GEE) was used to assess factors affecting the difference observed between FAZ measurements of different methods. A *P* value < 0.05 was considered significant.

## Results

In total, 126 eyes of 81 subjects and 37 eyes of 25 subjects were included in the training and testing group, respectively. The mean ± SD of corrected distance visual acuity (CDVA) was 0.30 ± 0.24 LogMAR (Snellen equivalent of 6/12) in diabetic patients. Scan quality was 8.47 ± 0.767 and 6.66 ± 1.50 in healthy and diabetic subjects, respectively (*P* value = 0.004). Table 1 shows baseline characteristics of the training and testing group. There were no statistically significant differences between  the two sets with respect to age, presence of diabetic retinopathy, manual FAZ area and scan quality. The total duration of training was 332 s.

**Table 1:** Baseline characteristics of training/validation and testing set.

Variable	Training/validation set	Testing set	*P* value
Eyes	126	37	
Age (mean ± SD), years	56.3 ± 14.6	51.89 ± 13.93	0.101*
Manual FAZ area (mean ± SD), mm^2^	0.425 ± 0.171	0.384 ± 0.160	0.185*
Diabetic retinopathy (no, %)	104 (82.5%)	27 (72.9%)	0.239†
Diabetic macular edema (no, %)	31 (24.6%)	19 (51.4%)	0.006†
Foveal cyst (no, %)	24 (19%)	16 (43.2%)	0.004†
Scan quality (mean ± SD)	6.9 ± 1.5	7.3 ± 1.4	0.127*

*FAZ* Foveal avascular zone.

*Student’s *t* -test.

^†^Chi-square test.

**Figure 2:**
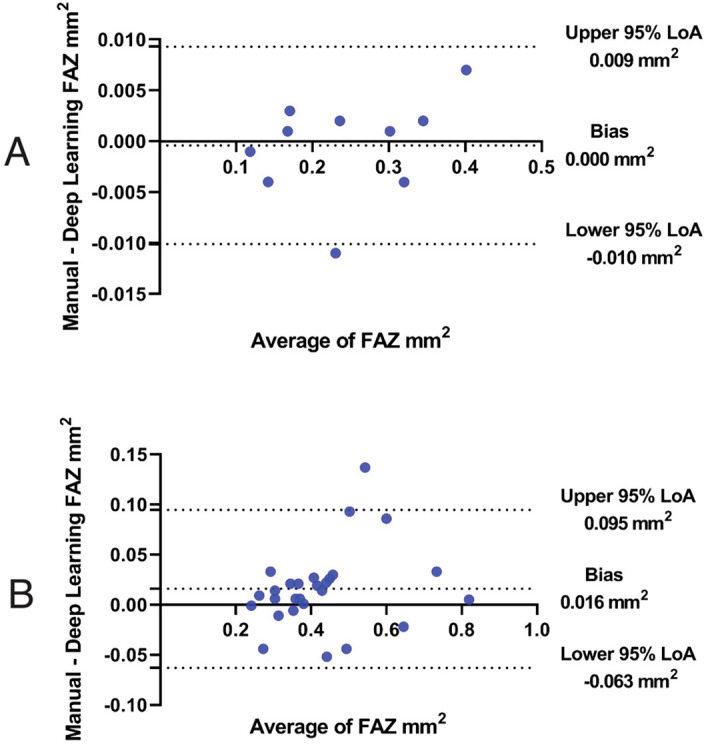
Bland–Altman plot demonstrates excellent agreement between manual and deep learning Foveal avascular zone (FAZ) segmentation in (**A**) normal and (**B**) eyes with diabetic retinopathy.

**Figure 3:**
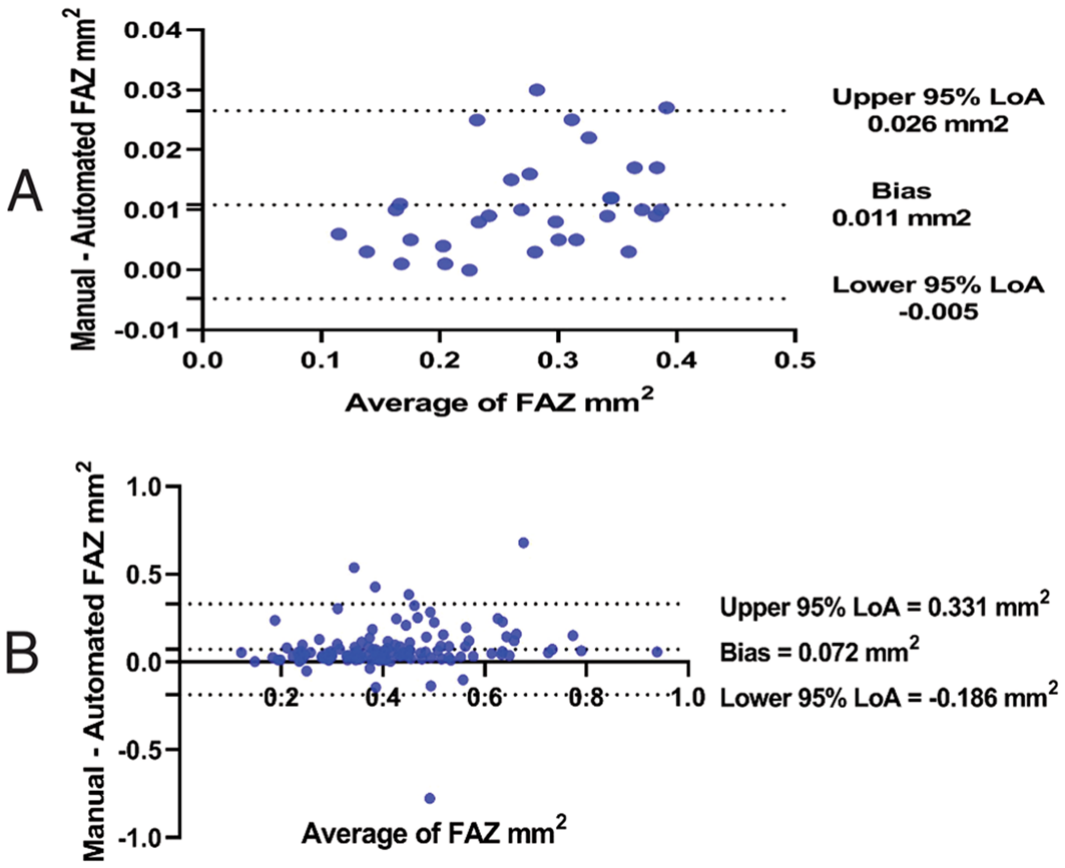
Bland–Altman plot shows (**A**) good agreement between manual and automated Foveal avascular zone (FAZ) area measurement in normal eyes, however, (**B**) poor agreement is evident in eyes with diabetic retinopathy.

**Figure 4:**
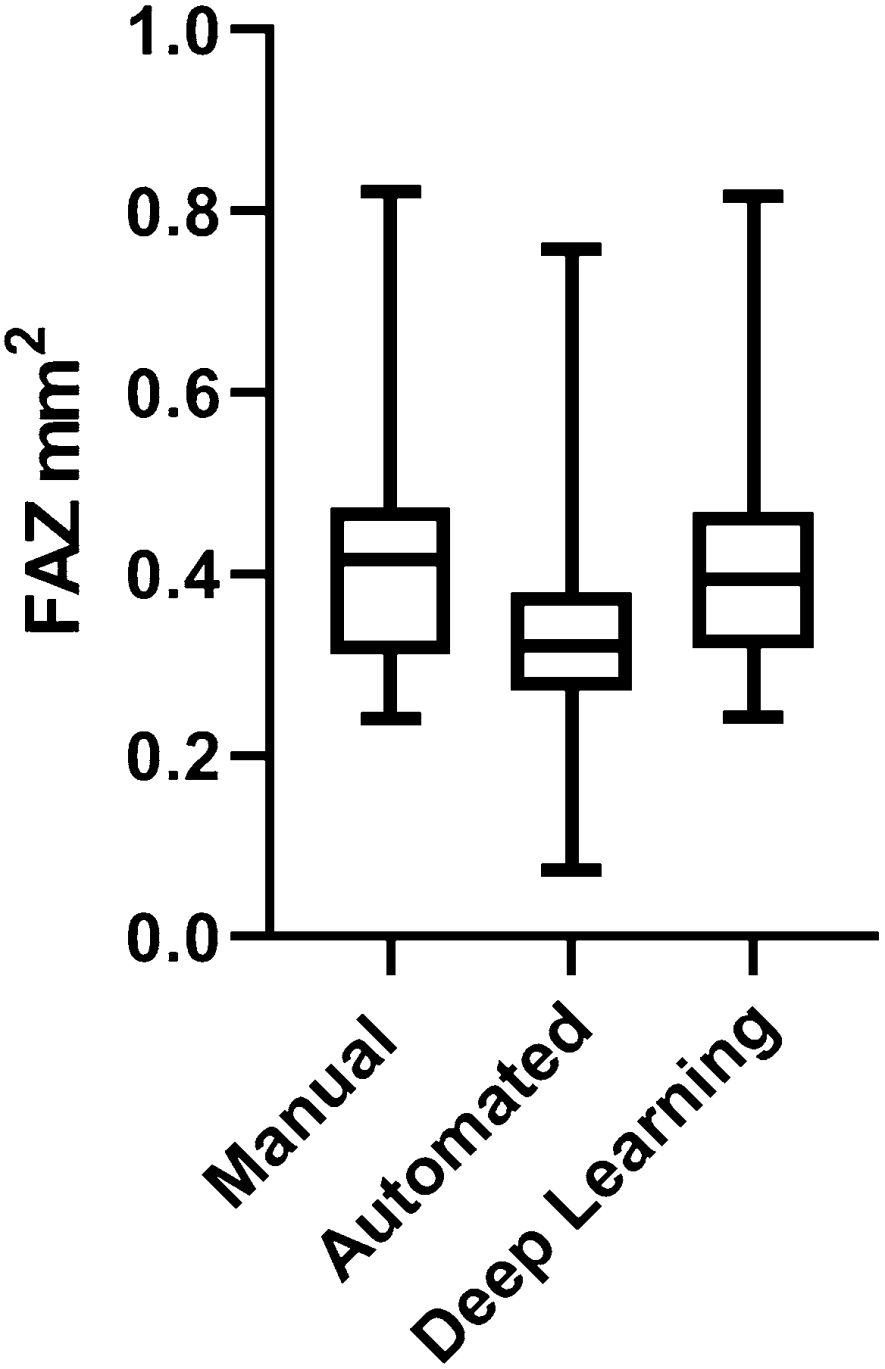
A bar and whisker plot shows the median and quartiles of foveal avascular zone size (FAZ), measured by three different methods. The automated FAZ size was significantly smaller than the areas measured by manual and deep learning methods (*P* value < 0.000).

**Figure 5:**
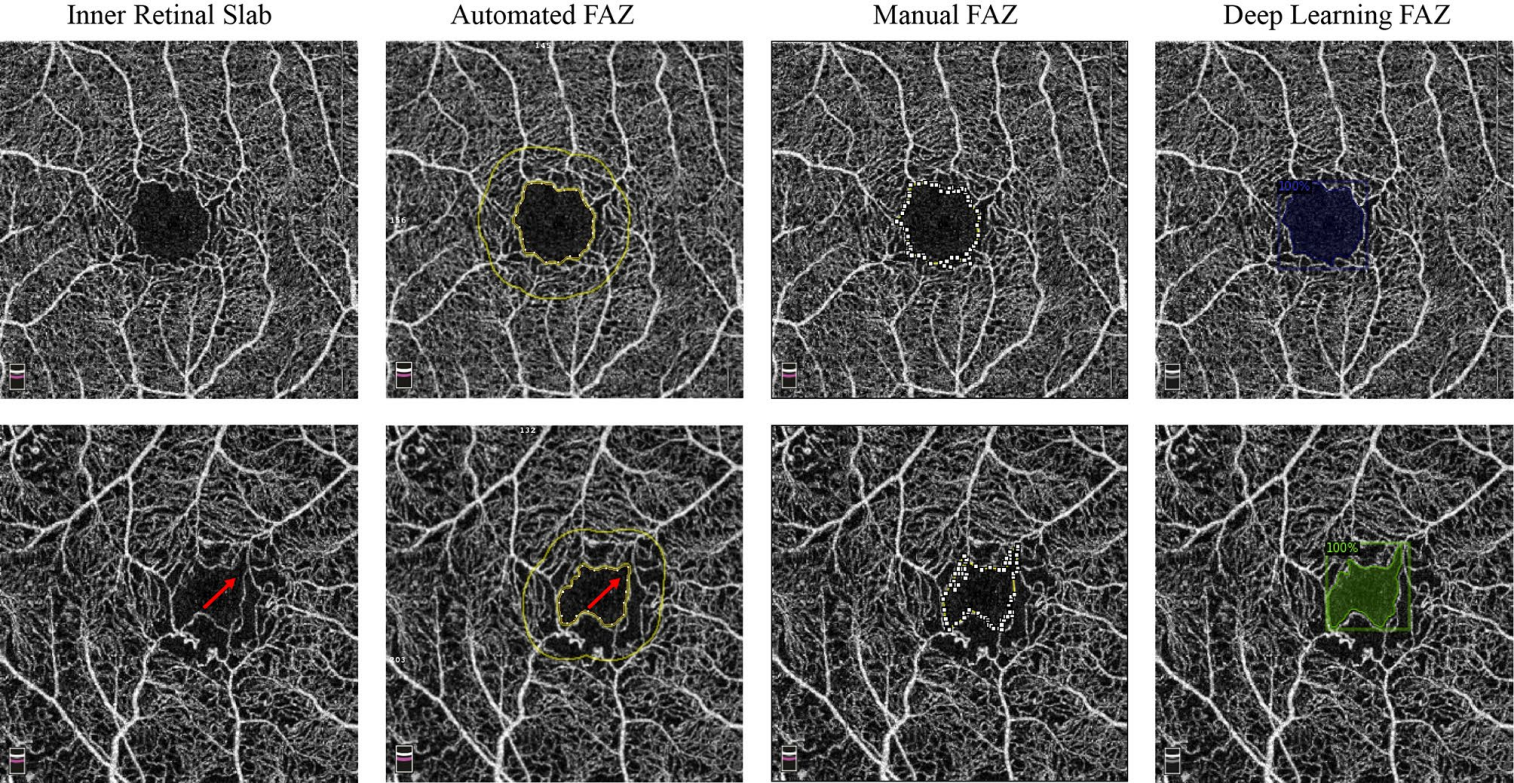
First row: the foveal avascular zone (FAZ) area measurements in a healthy subject were 0.337 mm^2^, 0.346 mm^2^ and 0.344 mm^2^ using automated, manual and deep learning methods, respectively. Second row: The foveal avascular zone (FAZ) area measurements in a diabetic patient were 0.219 mm^2^, 0.250 mm^2^ and 0.249 mm^2^ using automated, manual and deep learning methods, respectively. Note that the drawn line made by the built-in software (Red arrow) gives a false impression of correct FAZ delineation. (Python Software Foundation. Python Language Reference, version 3.6. Available at http://www.python.org).

The mean manual FAZ area was 0.282 ± 0.082 and 0.449 ± 0.168 mm^2^ in healthy subjects, and in diabetics (*P* = 0.000). The Bland–Altman analysis showed an excellent agreement between DL and manual FAZ measurements in testing group in both normal eyes and eyes with diabetic retinopathy. The mean differences between manual and DL FAZ area were 0.000 mm^2^ (95% LoA: − 0.010 to 0.009 mm^2^) and 0.016 mm^2^ (95% LoA: − 0.063 to 0.095 mm^2^) in normal eyes and eyes with diabetic retinopathy, respectively (Fig. 2). Although there was a good agreement between manual and automated FAZ measurements in normal eyes (Bias = 0.011 mm^2^, 95% LoA: − 0.005 to 0.026 mm^2^), a high variability was observed between manual and automated FAZ measurements in eyes with diabetic retinopathy (Bias = 0.072 mm^2^, 95% LoA: − 0.186 to 0.331, Fig. 3). The mean FAZ area was significantly lower in commercial measurement than DL and manual methods (Fig. 4). The correlation coefficient between manual and automated FAZ measurements was R = 0.996 and R = 0.652 in healthy subjects and eyes with diabetic retinopathy, respectively. However, the correlation coefficient between manual and DL FAZ measurements on the testing dataset was R = 0.995 and R = 0.962 in healthy subjects and eyes with diabetic retinopathy, respectively. Figure 5 illustrates an example of FAZ measurement using the 3 different techniques in healthy and diabetic subjects.

In univariate analysis, adjusted for inter-eye correlation, the absolute difference between manual and automated FAZ measurements was associated with age, diabetic retinopathy, diabetic macular edema and foveal cysts (Table 2). In multivariate analysis, diabetic retinopathy and presence of macular edema were significantly associated with absolute manual and automated FAZ size differences (β coefficient =  − 0.041, *P* value = 0.028 and β coefficient =  − 0.173, *P* value = 0.025, respectively).

**Table 2:** Summary of univariate regression model using absolute differences between manual and automated foveal avascular zone area as an outcome variable.

	β coefficient	*P* value *	95% CI
Age	0.002	0.004	0.001 to 0.003
Sex	0.005	0.793	− 0.036 to − 0.043
Diabetic retinopathy	− 0.080	0.000	− 0.103 to − 0.058
Diabetic macular edema	− 0.107	0.000	− 0.158 to − 0.054
Foveal cyst	− 0.075	0.002	− 0.127 to − 0.025
Scan quality	− 0.009	0.044	− 0.017 to – 0.001

*P values were calculated using generalized estimating equations to adjust for inter-eye correlations.

As for model metrics, the mean ± SD of DSC in the testing dataset was 0.94 ± 0.04. The mean ± SD of DSC was 0.97 ± 0.01 and 0.93 ± 0.04 in healthy subjects and eyes with diabetic retinopathy, respectively.

## Discussion

In this study, the agreement of the automated FAZ measurements conducted by the software of the RTVue instrument, manual measurements and a deep learning model developed by the authors was assessed. We considered the manual measurement as the gold standard technique for delineation of FAZ and compared those two methods with manual measurements. Our results showed that despite comparable measurements of FAZ area between the device automated software and manual method in healthy eyes (R = 0.996), the correlation was poor in diabetic patients (R = 0.652). On the other hand, the deep learning model exhibited excellent accuracy in detection of FAZ in both healthy subjects and diabetic patients (R = 0.995 and 0.962, respectively).

Although FAZ is usually a simple dark area in the center of the image in healthy subjects, the presence of various artifacts especially in diabetic eyes^16^ may interfere with the accuracy of available automated method as supported by our results. The AngioVue system uses an image processing technique for delineation of FAZ area. It seems that the artifacts and signal alterations caused by macular edema and intraretinal cysts in diabetic patients affect FAZ measurement made by the built-in software. This is in line with previous studies that reported higher rates of artifacts in eyes with retinal pathologies compared to healthy eyes^16^. The wide range of limit of agreement (− 0.186 to 0.331) in Bland–Altman plot comparing the automated FAZ measurement and manual technique shows that the automated FAZ measurements should be manually corrected in studies involving diabetic eyes. However, manual delineation of the FAZ is a time-consuming process (several minutes per image) needing trained image graders. On the other hand, the DL method offers a fast alternative for delineation of FAZ area (0.291 s/image vs. few seconds in the device's software). Therefore, our deep learning model is a promising alternative method especially in clinical trials with large sets of data.

Although a large body of literature is available regarding different image processing techniques for automatic delineation of FAZ area in various retinal imaging modalities^17–26^, studies focusing on FAZ segmentation in OCTA were usually conducted on healthy subjects (Supplementary Table S1). In addition, few studies assessing the accuracy of FAZ delineation in OCTA images of diabetic eye have failed to exhibit a high correlation (Intersection over Union: 0.70^21^ and 0.82^20^), due to high incidence of signal noise and artifacts in OCTA imaging of diabetic patients^16^.

Limited studies reported the use of deep learning in OCTA imaging. Guo et al.^27^ reported a fully convolutional deep learning model for FAZ measurement in superficial capillary plexus en face OCTA of healthy subjects. Their model provided a mean DSC of 0.976 which is comparable to DSC of 0.974 for delineation of FAZ in normal subjects of our study. In addition, our model was also trained for FAZ measurements in patients with diabetic retinopathy and in full retinal slab.

This study has some limitations. The sample size was small, and eyes with other retinal pathologies were not included in the study. However, the similarities between enface OCTA of eyes with DR and other ischemic retinal disorders might render the current model valid for different clinical situations. We could not analyze the results based on different stages of diabetic retinopathy due to the limited sample size and the quantitative data regarding macular edema was not available. Furthermore, we assessed accuracy of a single model, therefore our results might not be generalizable to other methods of deep learning (e.g. ResNet-50). Comparing different approaches in FAZ segmentation could be the subject of further research. In addition, our study was limited to images obtained by a single device, and in order to generalize the model to different devices, the model probably should also be trained on images exported from those devices.

In conclusion, our study showed that automated FAZ measurements made by the OCTA device’s built-in commercial software were comparable to the manual measurements in healthy subjects; however, the agreement was poor in diabetic eyes, especially in the presence of diabetic macular edema and intraretinal cysts. Deep learning model showed accurate FAZ delineation in both healthy subjects and diabetic eyes. Further studies with larger sample sizes and different retinal pathologies using different OCTA devices are needed to confirm our findings.

## Supplementary Information

Supplementary Information

## Data Availability

All data are available upon request.
