# Supplementary material for: Foveal avascular zone segmentation in optical coherence tomography angiography images using a deep learning approach
Source: Sci Rep. 2021 Jan 13;11:1031. doi: 10.1038/s41598-020-80058-x (PMC7806603; doi:10.1038/s41598-020-80058-x)
Supplement: Supplementary file 1 — Supplementary Information [file 41598_2020_80058_MOESM1_ESM.docx]

**Foveal avascular zone segmentation in optical coherence tomography angiography images using a deep learning approach**

**Authors:** Reza Mirshahi MD, MPH,^1^ Pasha Anvari MD, MPH,^1^ Hamid Riazi-Esfahani MD,^2^ Mahsa Sardarinia MD,^1^ Masoud Naseripour MD,^1,3^ Khalil Ghasemi Falavarjani MD* ^1,3^

1. Eye Research Center, The Five Senses Institute, Rassoul Akram Hospital, Iran University of Medical Sciences, Tehran, Iran.

2. Eye Research Center, Farabi Eye Hospital, Tehran University of Medical Sciences, Tehran, Iran.

3. Stem Cell and Regenerative Medicine Research Center, Iran University of Medical Sciences, Tehran, Iran.

**Correspondence to:** Khalil Ghasemi Falavarjani MD, Eye Research Center, Rassoul Akram Hospital, Sattarkhan-Niaiesh St, Tehran, Iran. TelFax : 0098-21-66509162, Email: [drghasemi@yahoo.com](mailto:drghasemi@yahoo.com)

**Competing Interest Statement:** None of the authors has any financial interest in the subject matter of this paper.

Table S1. Summary of studies reporting automated segmentation of foveal avascular zone in different imaging modalities.

| Study | Image Type | Subjects | Method | Metrics and Results | Limitations |
| --- | --- | --- | --- | --- | --- |
| Nugroho et al.^23^ | Color Fundus Image | Healthy and Diabetic (Messidor and DRIVE databases) | Image processing: matched filter and local entropy thresholding for segmentation of retinal vessels | Correlation coefficient: 0.912 and 0.802 in each database | Limited resolution of color fundus photos for delineation of FAZ area, Less than ideal performance in diabetic eyes |
| Agarwal et al.^24^ | Color Fundus Image | Healthy | Image processing: Difference of Gaussian (DoG) filter, Prewitt edge detection and dilation filters | Mean value: 673.04 ± 86.92 μm compared to 688.42 ± 72.18 in manual segmentation | Limited resolution of color fundus photos for delineation of FAZ area, Limited to healthy subjects, Not providing segmentation metrics |
| Hofer et al.^22^ | Fluorescein angiogram | Diabetic | Deep learning: Multi-task learning and Euclidean distance map | Dice Score: 0.805 | Less than ideal performance in diabetic eyes |
| Alipour et al.^19^ | angiogram | Healthy and Diabetic | Image processing: digital curvelet transform, morphological filters (dilation and erosion) and thresholding | IoU: 0.8553 ± 0.04 in normal and 0.8077 ± 0.1 in diabetic subjects | Less than ideal performance in diabetic eyes |
| Ishii et al.^18^ | Swept-Source OCTA:  superficial retinal layer | Healthy | Image processing: Kanno-Saitama Macro (KSM, ImageJ) by thresholding and morphological filters | Mean difference between the KSM and manual methods: 0.015 mm^2^ | Limited to healthy subjects, Not providing segmentation metrics |
| Lin et al.^25^ | OCTA: superficial retinal layer | Healthy | Image processing: KSM, , Level Sets macro (LSM) in ImageJ using partial differential equations, Cirrus built-in software | Dice coefficient: LSM: 0.9243, KSM: 0.9012, commercial software: 0.732 | Limited to healthy subjects |
| Carmona el al.^21^ | OCTA Images: superficial and deep plexus | Healthy and Diabetic | Image processing: template matching, adaptive binarization thresholding | Dice score: (0.70 ± 0.17) in complex dataset | Less than ideal in diabetic eyes |
| Diaz et al.^20^ | OCTA Images: superficial and deep plexus | Healthy and Diabetic | Image processing: Morphological operators (White top-hat), Canny edge detector, morphological closure (dilation) | IoU: 0.82 in healthy and 0.83 in diabetic patients | Less than ideal in diabetic eyes |
| Heisler et al.^26^ | OCTA Images | Healthy and Diabetic | Image Processing: FAZ area was calculated indirectly by defining it as the greatest avascular area after segmentation of retinal vasculature using deep convolutional neural networks | Mean FAZ area, Healthy: 0.280 ± 0.098 (manual) vs. 0.261 ± 0.080 (DL)  Diabetic: 0.594 ± 0.347(manual) vs. 0.492 ± 0.249 (DL) | Not providing segmentation metrics, Indirect assessment of FAZ area based on largest avascular area that might be misleading in diabetic patients |

IoU: Intersection over union; KSM: Kanno-Saitama Macro; Level Sets macro: LSM; FAZ: foveal avscular zone, DL: Deep learning, OCTA: Optical Coherence Tomography Angiography
